# Developing an upstream process for a monoclonal antibody including medium optimization

**DOI:** 10.1186/1753-6561-7-S6-P34

**Published:** 2013-12-04

**Authors:** Sevim Duvar, Volker Hecht, Juliane Finger, Matthias Gullans, Holger Ziehr

**Affiliations:** 1 Pharmaceutical Biotechnology, Fraunhofer Institute for Toxicology and Experimental Medicine (ITEM), Braunschweig, Germany

## Background

Monoclonal antibodies have been established as important therapeutics in cancer and autoimmune diseases. Hence, there is a growing interest in the production of monoclonal antibodies in pharmaceutical industry. In order to reduce timelines and costs of production the process and medium development is of central importance.

Perfusion processes are well known to achieve higher productivities compared with batch or fed batch. Major advantages of perfusion culture are that you can keep optimal culture medium conditions for the cells and realize higher performance. However, obtaining high performance requires the combination of process optimization as well as a well-balanced concentrated culture medium. Selecting the best system also depends on the shear sensitivity of the cell line, the robustness of the process and the scale used.

In upstream processing batch, fed batch and perfusion mode were applied. Design of Experiments (DoE) was used to develop a feed protocol for fed batch cultivations. In shake flask experiments the influence of temperature, osmolality, and pH to improve antibody yield was examined.

In a further study we compared different cell retention systems with regard to achieve high viable cell densities in a short time like required for a seed train application. The best results were achieved with the ATF system with cell densities up to 1.3 × 10^8 ^cells/ml and 4 fold improved product concentration compared to batch culture.

## Materials and methods

A CHO cell line producing the antibody G8.8 against Epithelial Cell Adhesion Molecule (Ep-CAM) was employed for the experiments performed in this study. The fermenters were Sartorius BBI Twin-System (2- and 5 L culture volume). We compared five different retention systems: SpinFilter (Sartorius BBI Systems), Cell Settler (Biotechnology Solutions), Centritech Lab III (Pneumatic Scale), Biosep (Applikon) and ATF (Alternate Tangential Flow; Refine Technology). The cell count was performed with CEDEX cell counter (Roche Diagnostics). The monoclonal antibody was quantified with HPLC-method using Protein A-column. Design of Experiments (DoE) was used to develop a feed protocol for Perfusion cultivations. In shake flask experiments we examined the influence of temperature, osmolality, and pH to improve antibody yield.

## Results

### Fed batch development in shake flasks with DoE

For the development of fed batch in shake flasks we used D-optimal Design with 18 runs. The examined factors were: Feed volume, time of feed start, time of temperature shift (33°C) and time of Osmolality shift (450 mOsmol/kg). The response was maximum antibody titer. The results show that the optimal feed volume is 15 ml/d. The time point for feeding start has almost no influence. The temperature shift and osmolality shift have negative influence (data not shown).

### Comparison of cultivations with different retention systems

We compared five different cell retention systems under same cultivation conditions. The best results could be achieved with the ATF system with cell densities up to 1.3 × 10^8 ^cells/ml. The next best retention systems were the Centrifuge and the Cell Settler with cell densities reached up to 3 × 10^7 ^cells/ml. Using BioSep and Spinfilter, cell densities up to 2 × 10^7 ^cells/ml were obtained (data not shown). The Spin filter and BioSep showed break through of cells at cell densities > 2 × 10^7 ^cells/ml. In contrast, the Cell Settler had the advantage of simplicity and robustness and no moving parts. The advantage of the centrifuge was the high flexibility concerning the reactor-volume to be perfused. The Spinfilter and BioSep showed the lowest performance.

### Comparison of cultivations with ATF

**Figure 1:**
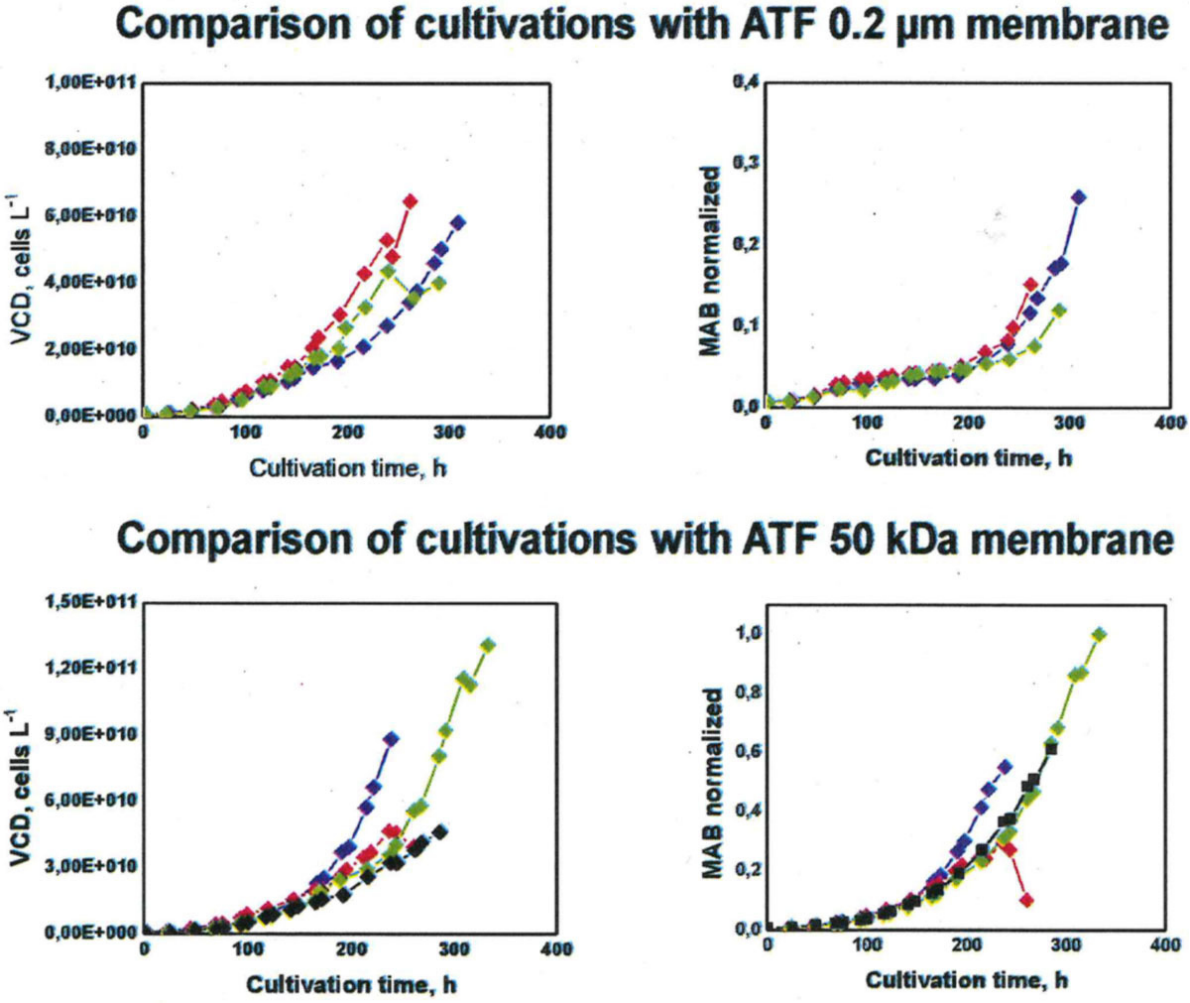
**Comparison of cultivations with ATF 0.2 μm and 50 kDa membrane**.

In a study we compared ATF cultivations with 0.2 μm membrane and with 50 kDa membrane. In cultivations with the 0.2 μm membrane a maximum cell density with 6.4 × 10^7 ^cells/ml could be achieved compared to a maximum cell density of 1.3 × 10^8 ^cells/ml with the 50 kDa membrane as shown in Figure 1. The increased cell densities resulted in a higher productivity compared to the other cell retention systems. Furthermore, the ATF with 50 kDa retended not only the cells but also the antibody within the reactor. Therefore, a higher volumetric productivity could be achieved with the 50 kDa membrane. The maximum titer in the reactor with the 50 kDa membrane was 4 fold higher compared with the 0.2 μm membrane.

Viable cell densities (VCD) and product concentrations of the monoclonal antibody (MAB) are shown.

## Conclusions

We have demonstrated that perfusion processes have a higher productivity compared to batch or fed batch processes. In our study the best retention system for perfusion culture was the ATF system compared with SpinFilter, Cell Settler, Centritech Lab III and Biosep. With the ATF system we realized cell densities up to 1.3 × 10^8 ^cells/ml and 4 fold improved product concentration compared to batch culture. Also, the ATF with a 50 kDa membrane retended not only the cells but also the antibody within the reactor. Therefore, a higher volumetric productivity could be achieved with the 50 kDa membrane. In perfusion culture the cells show constant specific productivity over the whole perfusion phase which shows that the cells are well fed.

